# Modulation of the Vault Protein-Protein Interaction for Tuning of Molecular Release

**DOI:** 10.1038/s41598-017-12870-x

**Published:** 2017-11-01

**Authors:** Kang Yu, Yin Hoe Yau, Ameya Sinha, Tabitha Tan, Valerie A. Kickhoefer, Leonard H. Rome, Hwankyu Lee, Susana G. Shochat, Sierin Lim

**Affiliations:** 1 Bioengineering Division, School of Chemical and Biomedical Engineering, Nanyang Technological University, 70 Nanyang Drive, Singapore, 637457 Singapore; 2 Structural Biology and Biochemistry Division, School of Biological Sciences, Nanyang Technological University, 60 Nanyang Drive, Singapore, 637551 Singapore; 3 School of Materials Science and Engineering, Nanyang Technological University, 50 Nanyang Avenue, Singapore, 639798 Singapore; 4 Department of Biological Chemistry, David Geffen School of Medicine at UCLA, Los Angeles, CA 90095 USA; 5 California NanoSystems Institute, University of California Los Angeles, Los Angeles, CA 90095 USA; 6 Department of Chemical Engineering, Dankook University, Jukjeon, Yongin 448-701 South Korea; 7 NTU-Northwestern Institute for Nanomedicine, Nanyang Technological University, 50 Nanyang Drive, Singapore, 637553 Singapore

## Abstract

Vaults are naturally occurring ovoid nanoparticles constructed from a protein shell that is composed of multiple copies of major vault protein (MVP). The vault-interacting domain of vault poly(ADP-ribose)-polymerase (INT) has been used as a shuttle to pack biomolecular cargo in the vault lumen. However, the interaction between INT and MVP is poorly understood. It is hypothesized that the release rate of biomolecular cargo from the vault lumen is related to the interaction between MVP and INT. To tune the release of molecular cargos from the vault nanoparticles, we determined the interactions between the isolated INT-interacting MVP domains (iMVP) and wild-type INT and compared them to two structurally modified INT: 15-amino acid deletion at the C terminus (INTΔC15) and histidine substituted at the interaction surface (INT/DSA/3 H) to impart a pH-sensitive response. The apparent affinity constants determined using surface plasmon resonance (SPR) biosensor technology are 262 ± 4 nM for iMVP/INT, 1800 ± 160 nM for iMVP/INTΔC15 at pH 7.4. The INT/DSA/3 H exhibits stronger affinity to iMVP (*K*_D*app*_ = 24 nM) and dissociates at a slower rate than wild-type INT at pH 6.0.

## Introduction

Vaults are large ribonucleoprotein cellular organelles (40 × 40 × 67 nm, 13 MDa) with a hollow, barrel-like interior with volume measuring ~5 × 10^4^ nm^3^ ^1–4^. Discovered in 1986 as a contaminant in a coated vesicles preparation, they are found to be ubiquitous in the cytoplasm of most eukaryotic cells, with exceptions include yeast, worm, and fruit fly^5^. The vault structure appears to be dynamic as observed in the “breathing”^2,6^ and half vault exchange^7^ in solution. Vaults have been found to be non-immunogenic and nontoxic^8,9^. In addition, they can be engineered to carry hydrophobic molecules, decorated with peptides for the attachment of antibody and targeting to specific cell receptors as well as endosomal escape, making vaults promising as nanocapsules in drug delivery applications^9–14^.

The major vault protein (MVP) contributes up to 70% of the total mass of the vault, while poly(ADP-ribose)-polymerase (VPARP), telomerase-associated protein 1 (TEP1)^15^ and several copies of non-protein-coding vault RNA (vRNA)^5,16^ are found to associate inside the particles making up the remaining mass. Multiple MVPs (78 copies) assemble on polyribosomes during their synthesis to form vault-like structure *in vivo* ^17–19^. Even more interesting, modifications on MVP have been reported as potential biomarkers for many types of tumors such as: breast cancer, myeloid leukemia, non-small-cell lung cancer, glioblastoma^20–23^. A domain located at the C-terminus of VPARP (INT, GenBank accession No. AF158255; residues 1563–1724) interacts with MVP specifically near the waist of the vault, at the luminal side.

Leveraging on the specific interactions between INT and MVP, the INT domain can serve as a shuttle to transport molecules into the cavity of the vault^24^. Protein-based therapeutic molecules can be fused to the N-terminus of INT. We hypothesize that modification of this interaction by truncation or surface engineering of INT will result in modulated binding and release profile. To characterize and study the interaction, we have isolated INT-interacting MVP domains (iMVP) which bind to the INT. Our study provides a new insight into tuning the release of molecular cargo from the lumen of the vault through modulation of the interaction between iMVP and INT.

## Results and Discussions

### Predicting iMVP-INT interaction

**Figure 1:**
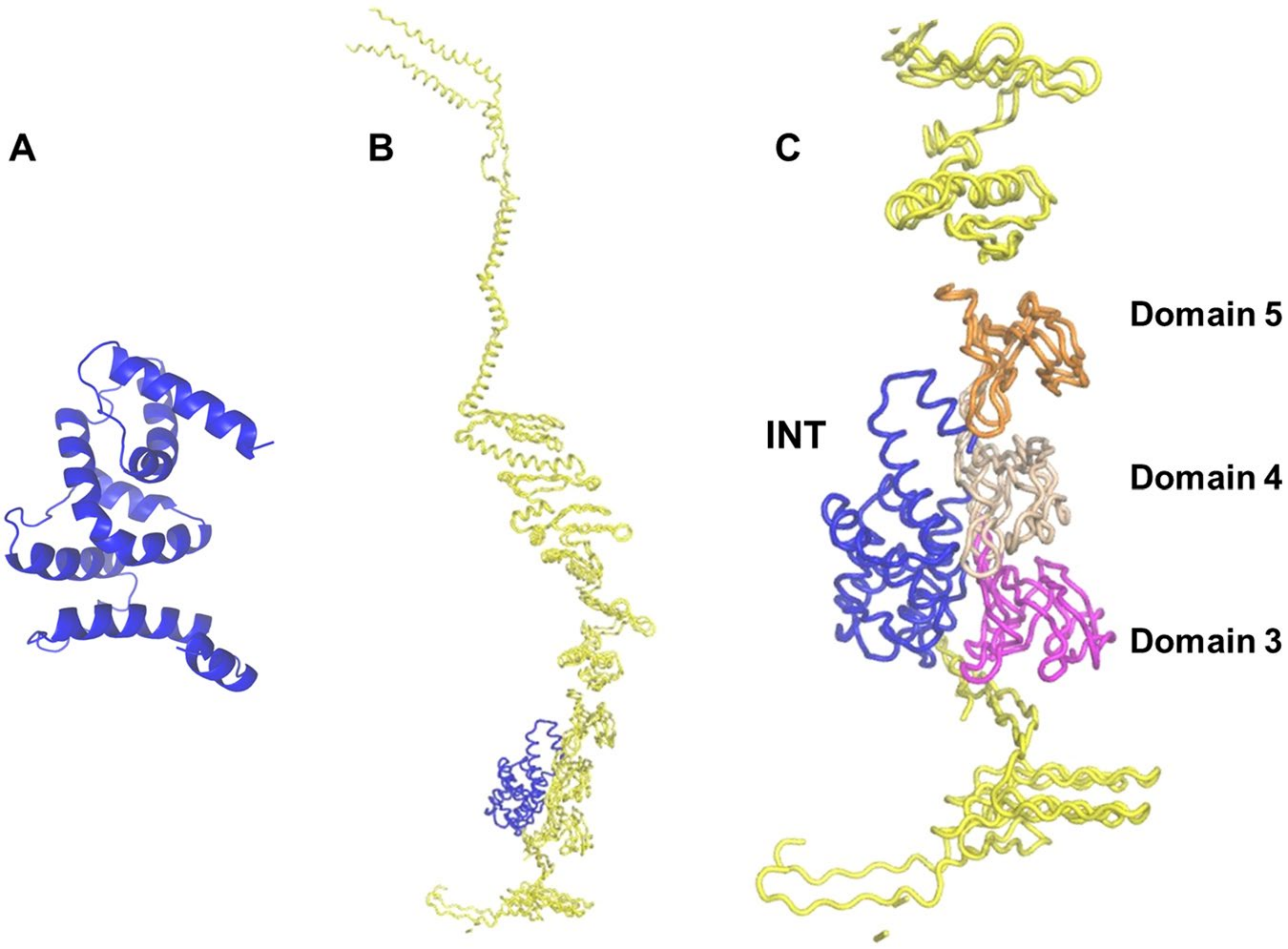
Prediction of INT structure and iMVP-INT interaction. (**A**) Predicted structure of INT using I-TASSER online portal. (**B**) Docking model of CP-MVP (yellow) and INT (blue) using GRAMM-X protein docking web server. (**C**) A close-up of the iMVP domains 3 (magenta), 4 (wheat), 5 (orange) and INT (blue).

The MVP domains that interact with INT have been identified. Cryoelectron microscopy (cryo-EM) difference mapping showed that luciferase-tagged INT (luc-INT) localizes in the barrel at positions above and below the waist^25^. To map the interaction at the critical region, the I-TASSER-predicted INT tertiary structure was docked to CP-MVP (Protein Data Bank ID 2QZV^25^) on the GRAMM-X web server^26,27^. In our model (Fig. 1), INT is predicted to dock at the lumen side of MVP, ranging over MVP domains 3, 4, and 5 indicating that the iMVP is located between domain 3 and 5. This prediction is consistent with the NMR results by Kozlov and co-workers^28^. In their spectra, the VPARP is revealed to have an obvious interaction with MVP repeat domains 3 and 4.

### Confirming interactions between isolated iMVP and INT

To investigate the interaction between iMVP and INT, we isolated the specific MVP domains 3 to 5 (PDB ID 2QZV, residues 102–276, including the gap between domains 2 and 3; MVP domain partitions can be found in Supplementary Information Table S1). The isolated domains 3 to 5 are not expected to form the vault barrel-like structure because iMVP is devoid of other MVP domains that interact to form the waist (domains 1 and 2) and the caps (domains 6 to 14). Histidine-tagged iMVP (His-iMVP) and mCherry-tagged INT (mCherry-INT) were produced recombinantly in *E. coli*. The preliminary interaction was confirmed using affinity chromatography (see Supplementary Information Fig. S1 for more details).

**Figure 2:**
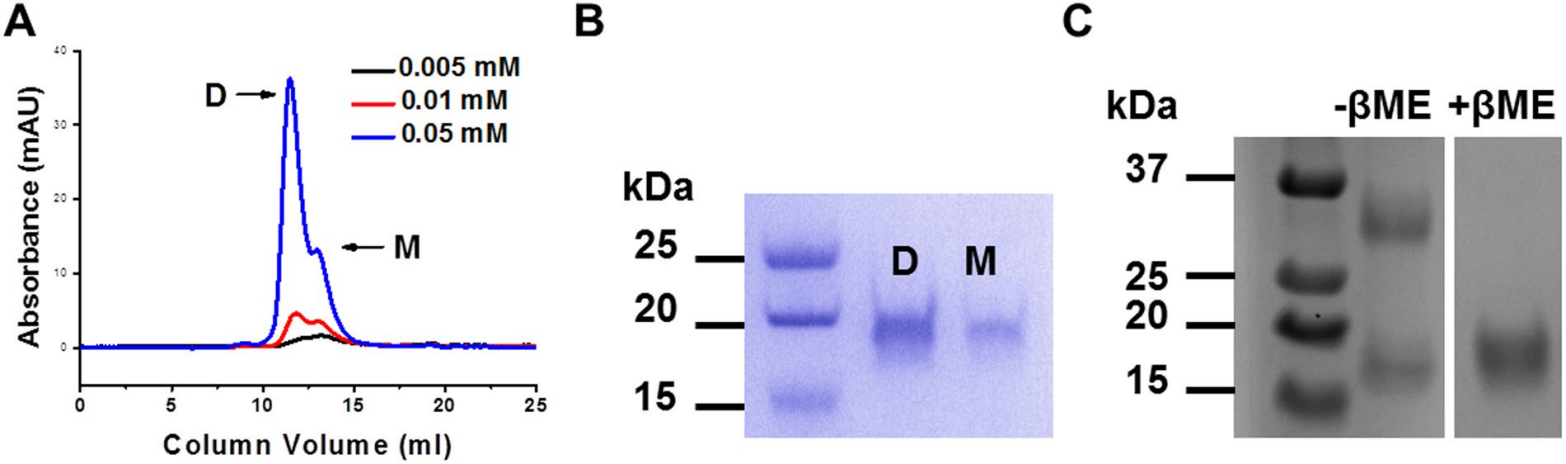
(**A**) SEC profile of His-INT of 0.05, 0.01 and 0.005 mM. The molecular mass of D and M was calculated to be 35.4 and 19.9 kDa, respectively. (**B**) SDS-PAGE of the D and M fractions from SEC of His-INT. Both fractions share similar molecular mass following reduction with β-mercaptoethanol (βME). (**C**) SDS-PAGE of the D fraction without (−βME) and with (+βME). Only partial SDS-PAGE images are shown on (**B** and **C**); see Supplementary Information Fig. S2 for full SDS-PAGE gel.

In subsequent experiments, iMVP and INT were produced as His-tagged proteins for ease of purification. Prior to the interaction study, the individual proteins were subjected to size exclusion chromatography (SEC). The His-iMVP molecular mass was determined to be 35.4 kDa, deviating from the result of mass spectrometry (20.8 kDa). The discrepancy may be due to the elongated fibrous shape of the His-iMVP, while the SEC size estimation assumes the proteins to have globular shape. The SEC profile shows that His-INT exists as both monomer and dimer with increasing dimer proportions at increasing His-INT concentration (Fig. 2A,B). The M fraction, with higher proportion of His-INT monomer, was used in the characterizations.

The molecular mass of the monomeric His-INT protein determined using SEC is in agreement with the calculated value of 20.76 kDa based on the amino acid sequence. Molecular mass determination of the SEC fractions using MALDI-TOF/TOF mass spectrometry (MS) suggests that His-INT also forms dimer (41.2 kDa; more detail can be found as Supplementary Information Fig. S3). This observation is due to the presence of three cysteine residues (GenBank accession No. AF158255; residues 1563, 1622 and 1687) on the His-INT (Fig. 2C). In all subsequent experiments and calculations, the M fractions have been assumed to be monomeric. This assumption is based on the presence of predominantly monomeric species while the monomer and dimer are in thermodynamic equilibrium at the given concentration (Fig. S4).

**Figure 3:**
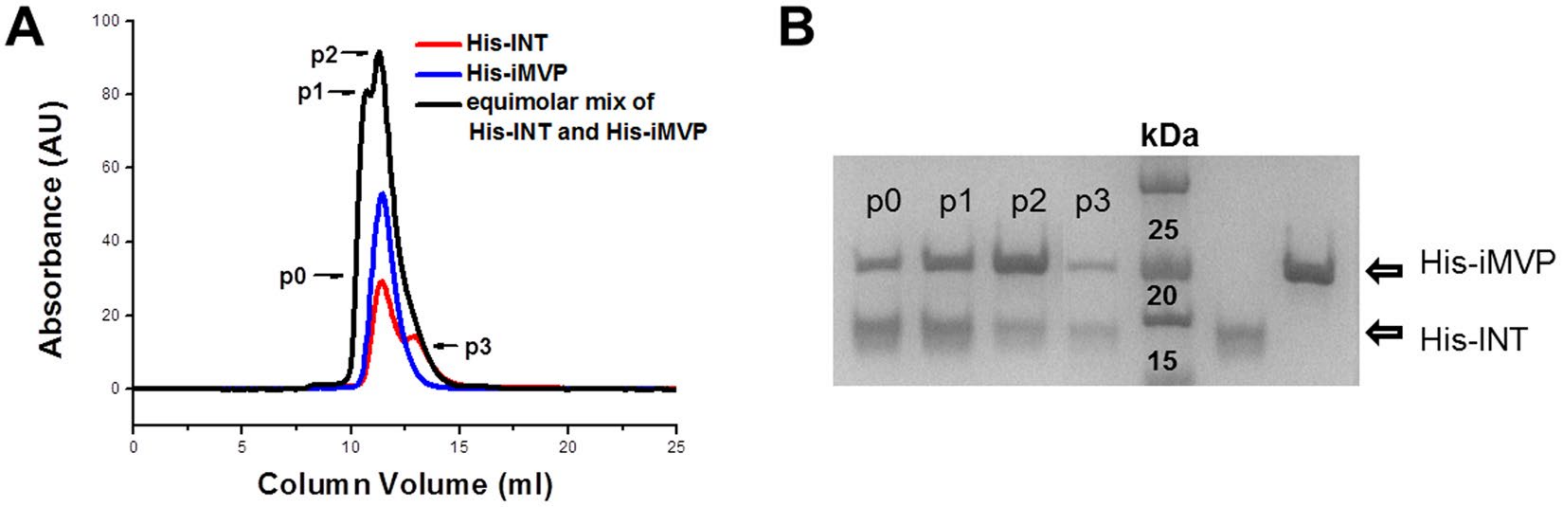
(**A**) SEC profile of equimolar mixture of His-iMVP and His-INT overlay single proteins. (**B**) SDS-PAGE gel of peak fractions from SEC in (**A**) (partial and merged; see Supplementary Information Fig. S5 for full SDS-PAGE gel).

To further analyze the interactions, pre-mixed His-iMVP and His-INT at equimolar ratio was loaded on the SEC column. At the working concentration of 70 μM, His-INT was assumed to be monomer during calculation. Instead of the expected single peak, two peaks are observed in the elution profile (Fig. 3A). Based on the calculated molecular mass from SEC data and SDS bands density ratio (Fig. 3B), the first obvious peak p1 on Fig. 3A (45.2 kDa) could be attributed to 1-to-1 complex (expected at 42.75 kDa). The calculated molar ratio of fraction p0 (1:1.8) in the gel suggests that there is a covered peak (p0) of His-iMVP and His-INT interacting at 2 INT to 1 iMVP in the eluted fraction. The band corresponding to His-iMVP is thicker than His-INT in fraction p2 with density ratio of 1:0.4, which indicates that the second obvious peak consists of predominantly His-iMVP protein. The shoulder at the tail of the 1-to-1 mixture elution profile (p3) shows overlapping peak of His-INT and His-iMVP. Based on the SEC data, His-INT interacts with His-iMVP at a ratio other than 1:1. A higher ratio (such as 1:3 or 2:2) is unlikely, due to the absence of elution peak at ≥ 84.3 kDa (the resolution range of the column was 3–75 kDa with void volume of 8 ml). Hence, the most probable ratio of iMVP:INT is 2:1 or 1:2 (the expected elution volume was 9.83–9.88 ml). The band density analysis of the SDS-PAGE gel (Fig. 3B) suggests that the ratio of interaction between iMVP and INT is most likely to be 1 iMVP to 2 INT as His-INT are present as both monomer and dimer at equilibrium.

### Characterization of iMVP-INT interaction

**Figure 4:**
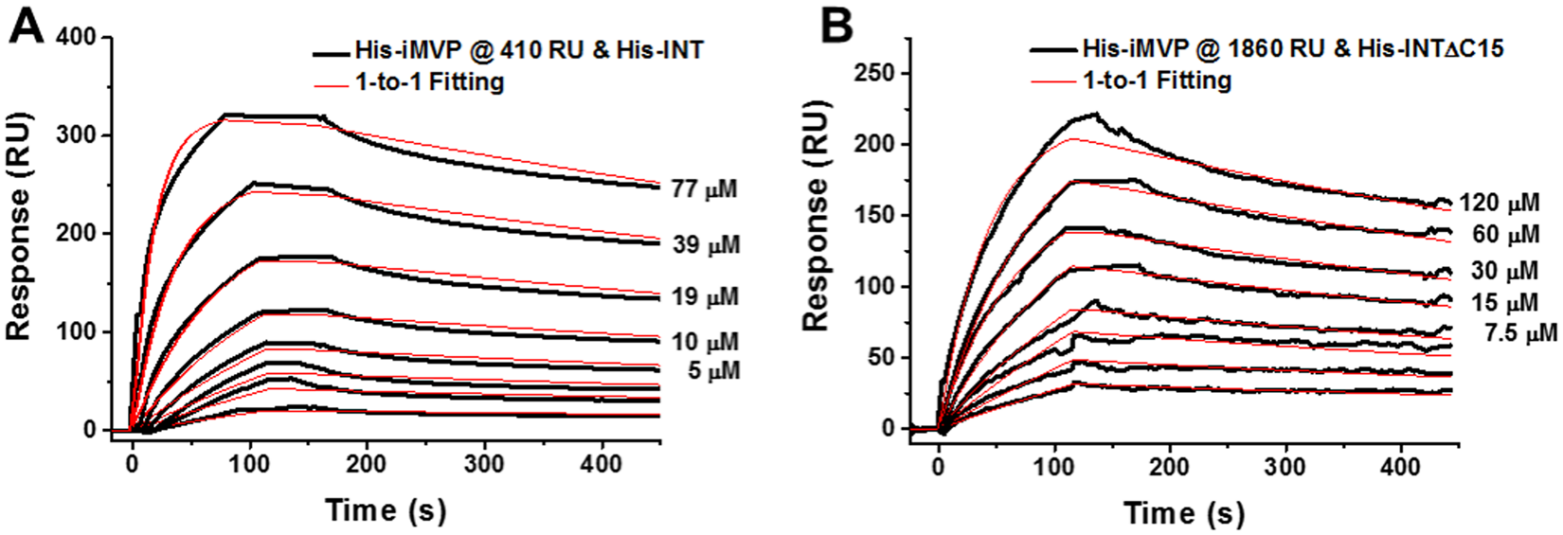
SPR sensorgrams depicting the interaction between His-iMVP and either (**A**) His-INT, with the lower three concentrations of 2.5, 1.2, and 0.6 µM, or (**B**) His-INTΔC15, with the lower three concentrations of 3.7, 1.8, and 0.9 µM. His-iMVP was immobilized at 410 RU for (**A**) and at 1860 RU for (**B**). The data were fitted to 1-to-1 Langmuir binding model.

To determine the binding strength between iMVP and INT, the interaction was studied using surface plasmon resonance (SPR) biosensor technology. The His-iMVP was immobilized on the surface of a CM5 sensor chip at 410 RU (1000 RU corresponds to 1 ng/mm^2^). Subsequently, His-INT was injected across the surface of the chip with concentrations ranging from 0.6 to 77 μM at 25 °C. The injections were repeated and the responses were superimposed. Figure 4 shows the sensorgrams of the SPR experiments at pH 7.4 in phosphate buffer. For His-INT, the curve fit follows 1-to-1 Langmuir binding model (red line) suggesting that His-INT binds to His-iMVP at a 1-to-1 ratio (Fig. 4A; Supplementary Information Figs S6 and S7 for other immobilization density). Under this condition, His-INT binds to His-iMVP with a dissociation rate constant (*k*_*d*_) of 6.0 × 10^−4^ s^−1^, which means half of the complex (iMVP-INT) would dissociate in 15 minutes (T_1/2_) in the running buffer (50 mM PBS, 150 mM NaCl, pH 7.4) at room temperature. Recognizing that the oligomeric state may change over the concentration range and the monomer and dimer are present in equilibrium at a given concentration, the affinity constant is referred to as apparent affinity constant (*K*_D*app*_) and not absolute affinity. The reference is reasonable since the fitting still fits the 1-to-1 Langmuir binding model. The *K*_D*app*_ for iMVP-INT was determined to be 262 ± 4 nM at pH 7.4 by fitting to the standard curve, with an association rate constant (*k*_*a*_) of 2280 M^−1^s^−1^. The range of *K*_D_ values for the strongest antibody-antigen affinity is 1 pM–10 nM. In contrast, transient protein-protein interactions have a *K*_D_ of higher than 10^−6^ M^29^. For example, interactions with a *K*_D_ of 6.2 × 10^−5^ M such as that of E-selectin-ESL-1 indicates transient interaction^30^. Comparison of the *K*_D_ values suggests that the interaction of iMVP-INT approaches that of medium-affinity antibody-antigen binding.

Goldsmith *et al*. reported that INT binds to recombinant vaults despite a 15-amino acid deletion from the C-terminus^24^. We hypothesize that truncation of INT will alter the interaction and the molecular release rate of packaged materials from the vault. To modulate the release rate based on our hypothesis, His-INTΔC15 (derived from VPARP residues 1563–1709, with 15 amino acids deleted at the C terminus of His-INT) was constructed and the interaction between His-iMVP and His-INTΔC15 was also studied by SPR. The His-INTΔC15 at concentrations ranging from 0.94 to 120 μM was injected over His-iMVP immobilized at density of 1860 RU. The results show that the His-INTΔC15 binds to His-iMVP at 1:1 with affinity constant of 1.83 μM (*k*_*a*_ ~ 490 M^−1^s^−1^, *k*_*d*_ ~ 8.9 × 10^−4^ s^−1^) (Fig. 4B). Without the 15 amino acids at the C terminus, His-INTΔC15 showed faster dissociation from His-iMVP than full-length His-INT and slower association (Table 1), confirming that it is possible to tune the release by modifying the length of INT domain.

**Table 1:** Comparison of Affinities of His-iMVP and His-INT at pH 7.4.

	*k*_*a*_ (M^-1^s^-1^)	*k*_*d*_ (s^-1^)	T_1/2_ (min)	*K*_D*app*_ (nM)
His-INT	2280	6.0 × 10^−4^	13.9	262
His-INTΔC15	485	8.9 × 10^−4^	9.4	1800

### Tuning the release profile by modifying the interaction surface

So far, we have determined the interactions between His-iMVP and His-INT and between His-iMVP and His-INTΔC15 where the latter is weaker. As an alternative approach to tune the release of molecules, we took a rational design approach by identifying several key amino acids located on the interacting surface on INT and substituting them with histidines. More design information of His-INT/DSA/3H can be found in Table S3 (Supplementary Information). We have previously demonstrated that placing a group of histidines strategically on E2 protein cage induces repulsive interactions between protein subunits upon pH change from 7.4 to 5.0 ^31,32^. The histidine imidazole side-chain has a pKa value of 6.1^33^. Upon pH change from 7.4 to 5.0, multiple protonated histidines that are strategically located within the Debye radius at the interface between proteins will induce enough repulsive forces to trigger disassembly or separation between protein subunits. In contrast, in our current study, we find that the protonation of histidines at pH 6.0 enhances the binding of INT to MVP. A molecular docking model shows that the key interaction sites on iMVP are negatively charged which explains our results. Upon protonation of histidines at pH 6.0 on INT, the interaction could be enhanced by electrostatic interaction. This mutation may assist in retention of the molecular cargo within the vault lumen in acidic microenvironment. The retention characterized here is expected to extend the release of molecular cargo from the vault lumen.

**Figure 5:**
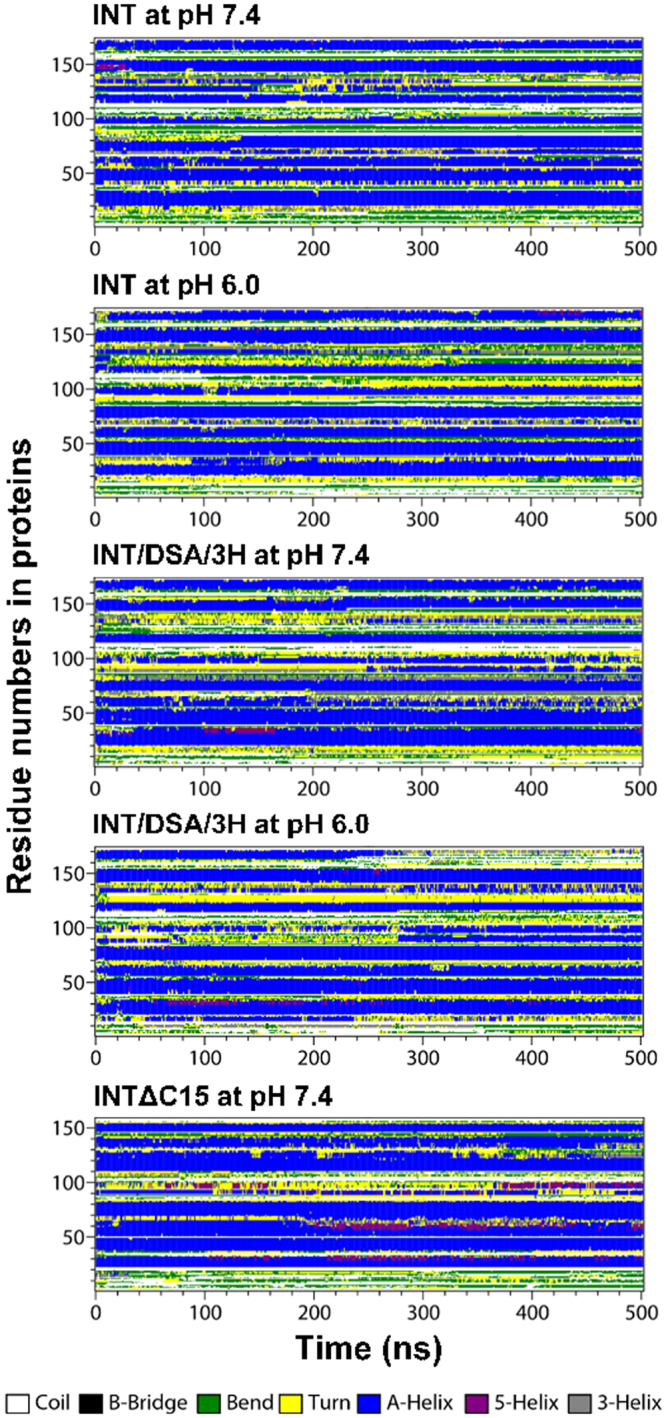
Secondary structure profiles of the protein as a function of time.

**Figure 6:**
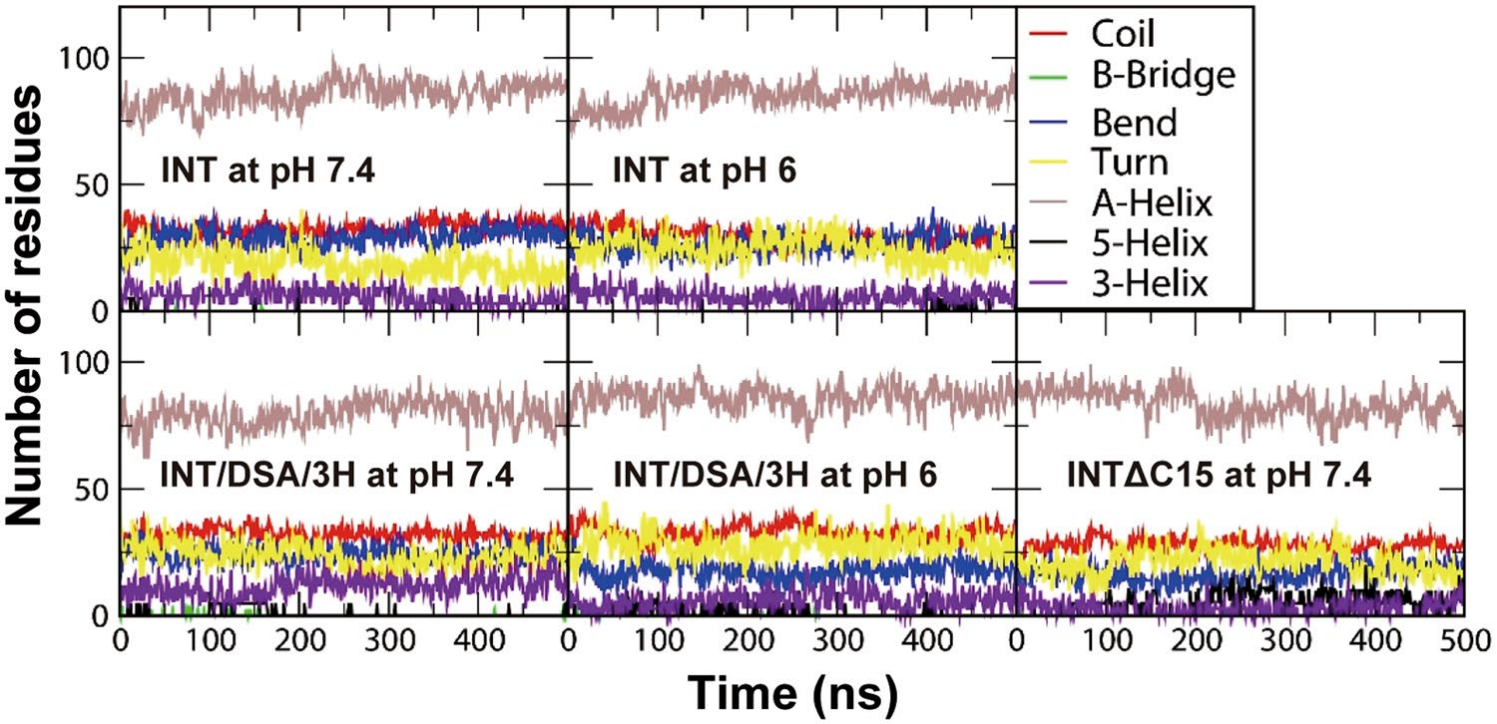
Numbers of residues for different structures in the protein as a function of time.

To understand the effects of protein sequence and pH condition on the protein structure, INT, INT/DSA/3H, and INTΔC15 were simulated at two levels of protonation to mimic their electrostatic charges at pH 7.4 and 6.0, and their secondary structures were calculated using the DSSP program^34^. Fig. 5 shows that proteins retain various structures over the whole simulation time, while the helical structure is much more prominent. To quantify this, we also calculated the number of residues for different structures as a function of time. In Fig. 6, the numbers of residues reach steady-state values with in 300 ns, indicating that simulations are well equilibrated. The numbers of α-helical residues of INT, INT/DSA/3 H, and INTΔC15 are 88 (±1), 81 (±2), and 82 (±1), respectively, at pH 7.4, indicating no significant effect of protein sequences on the protein structure. At pH 6.0, the numbers of α-helical residues of INT and INT/DSA/3H are both 86 (±1), similar to those at pH 7.4, indicating no dependence on the protonation state of His (pH). Other structures of coil, bend, and turn are also similarly observed in all simulated systems. These simulations show that the secondary structures of INT, INT/DSA/3H, and INTΔC15 are similar and are independent of the protonation state of His (pH). The findings indicate that the protein sequence and the His-protonation state do not significantly influence the protein structure, implying that the difference in binding affinity of INT, INT/DSA/3H, and INTΔC15 to His-iMVP is likely due to charge.

**Figure 7:**
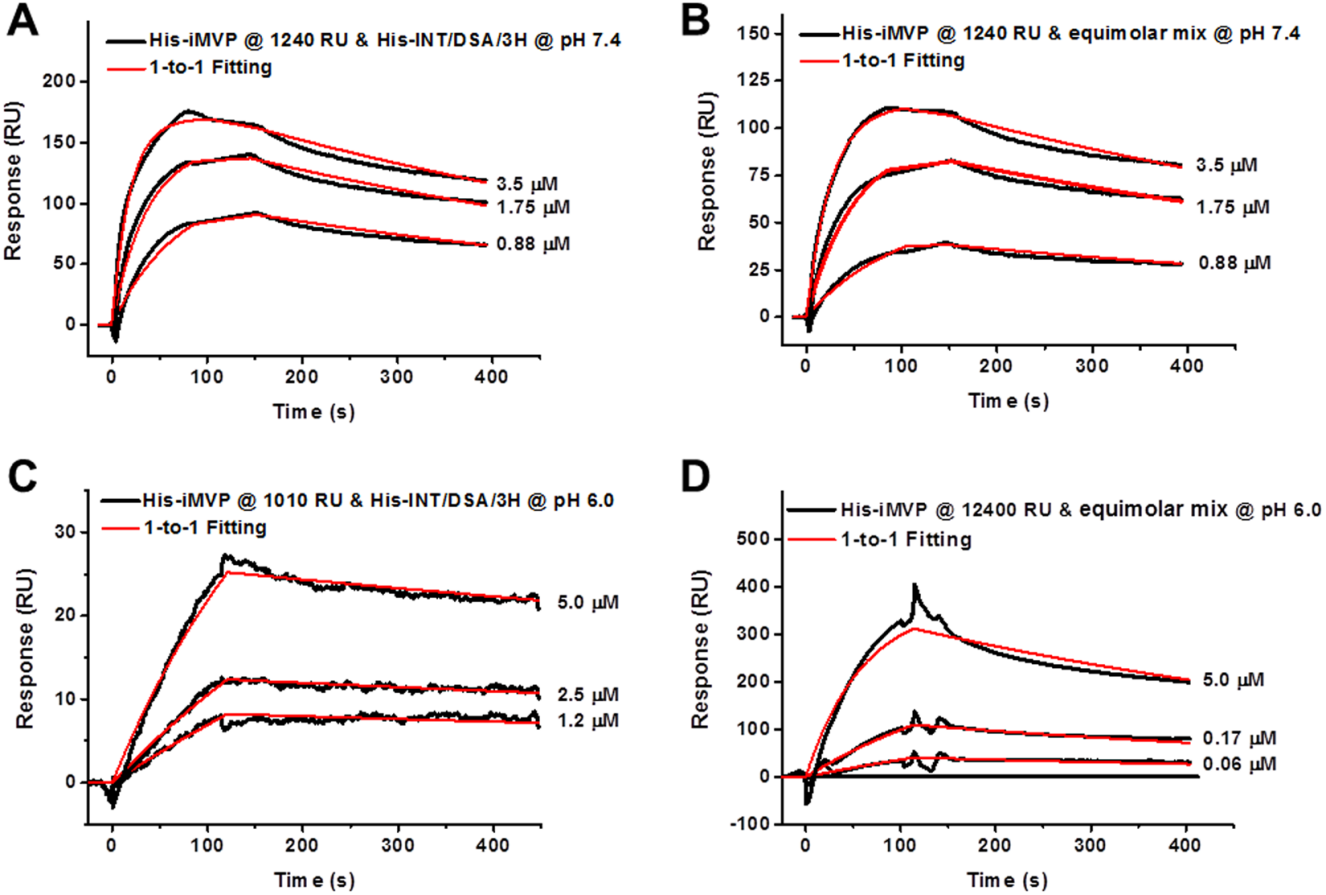
SPR sensorgrams of His-iMVP (immobilized at 1240 RU) and mobile phase at pH 7.4 (**A**,**B**) and 6.0 (**C**,**D**). (**A**,**C**) His-INT/DSA/3H, (**B**,**D**) equimolar mixture of His-INT and His-INT/DSA/3H.

The interaction between His-iMVP and His-INT/DSA/3H was also studied by SPR and the biophysical parameters were determined. At His-iMVP immobilization density of 1240 RU, His-INT/DSA/3H (at concentrations 0.88, 1.75, or 3.5 μM) was injected across the surface at pH 7.4. Subsequently, the bound complex was dissociated in the running buffer at the same pH. The mutant His-INT/DSA/3H shows 6.5 times faster binding to His-iMVP compared to His-INT, while the dissociation from His-iMVP is also faster compared to His-INT at 2.2 times. The affinity of His-INT/DSA/3H towards His-iMVP is higher than for the wild-type His-INT as reflected by *K*_D*app*_ of 90 nM (Fig. 7A). Tuning the dynamic balance between association and dissociation of His-iMVP and His-INT/DSA/3H could be used as a strategy for controlling the release rate of potential therapeutic molecules from the vault lumen. In this context, we hypothesized that combining the lower affinity binding of His-INT with the higher affinity binding His-INT/DSA/3H will result in an intermediate release rate. His-INT and His-INT/DSA/3H were mixed at equimolar ratio (in a series of concentrations 0.88, 1.75, or 3.5 μM) to study the association and dissociation with His-iMVP using SPR. The data were analyzed using a 1-to-1 Langmuir model without further deconvolution for comparison even though we are aware that the interaction recorded is a combination of two different components. The results of the competition between His-INT and His-INT/DSA/3H show that the *K*_D*app*_ between His-iMVP and the mixture is 114 nM at pH 7.4 (Fig. 7B).

During the mixture injection, the presence of more positive charges on His-INT/DSA/3H than wild-type His-INT results in preferred binding to His-iMVP. It is important to note that the affinity constant of 114 nM between His-iMVP and the His-INT/(His-INT/DSA/3H) mixture does not refer to average *K*_D*app*_ of the individual equimolar bound component. The values reflect *K*_D*app*_-s and only serve as a reference.

We subsequently showed that the binding affinity of His-INT could be further tuned at pH 6.0 by mixing His-INT with His-INT/DSA/3H in equal ratio. An interesting phenomenon was observed on His-INT/DSA/3H binding to His-iMVP at immobilization density of 1010 RU (Fig. 5C). His-INT/DSA/3 H was injected across the surface in a series of concentrations (i.e. 0.12, 0.25, or 0.5 μM). The dissociation rate of the captured His-INT/DSA/3H was very slow and the *K*_D*app*_ was calculated to be 24 nM. The interaction between His-iMVP and His-INT/DSA/3H is stronger than that between His-iMVP and His-INT at pH 6.0 (Fig. S8, Supplementary Information). Equimolar mixture of His-INT and His-INT/DSA/3 H (in a series of concentrations 0.06, 0.17, 0.5 μM) showed 1-to-1 binding to His-iMVP immobilized at 12400 RU and the *K*_D*app*_ was determined to be 79 nM at pH 6.0 (Fig. 5D). Experiments with His-iMVP at pH 6.0 were inconclusive due to its non-specific binding to the chip.

His-INT/DSA/3H shows stronger affinity to His-iMVP than the wild-type His-INT (Table 2). Vaults exhibit a more open conformation at low pH^35^. With the modulated protein-protein interaction, the molecular cargos could be better retained hence, reducing the burst release during the transient open state.

**Table 2:** Comparison of affinities of His-INT variants at pH 7.4 and 6.0.

pH	Mobile phase	*k*_*a*_ (M^−1^s^−1^)	*k*_*d*_ (s^−1^)	*K*_D*app*_ (nM)
7.4	His-INT/DSA/3H	14900	1.34 × 10^−3^	90
	Equimolar Mixture*	10800	1.23 × 10^−3^	114
6.0	His-INT/DSA/3H	10800	2.58 × 10^−4^	24
	Equimolar Mixture*	18500	1.47 × 10^−3^	79

His-INT/DSA/3H is more competitive than His-INT. The affinity constant of the mixture shows as intermediate values of His-INT and His-INT/DSA/3H and not the average. ^∗^Equimolar mixture is defined at injection.

## Conclusions

The predicted docking shows that the INT interaction sites are located between MVP domains 3 and 5. The SEC profile of His-INT shows that dimer and monomer of His-INT are present in equilibrium. As determined by SPR technique, His-INT binds to His-iMVP in a 1:1 interaction with an apparent affinity constant of ~260 nM. It should be noted that this study has examined only the interactions between His-iMVP and His-INT at 25 °C and pH 7.4.

The introduction of histidines to His-INT results in stronger interaction between His-iMVP and His-INT/DSA/3 H compared to the wild-type His-INT at both pH 6.0 and 7.4. The modification should allow longer retention of molecular cargo within the vault hollow core and results in a slower release rate. Mixing His-INT and His-INT/DSA/3 H shows an intermediate release rate. This study implies that modulation of molecular release rate from the vault lumen should be possible by tuning the proportion of wild-type and histidine-substituted INT or truncation of the INT. The physical characterization of iMVP-INT interaction is the first step towards designing modulated molecular cargo release from the vault lumen.

## Material and Methods

*E. coli* DH5α was obtained from Zymo Research and BL21(DE3) was from Stratagene. LB medium, vectors pET-11a and pET-28a were from Novagen. Columns for protein purification and sensor chip CM5 were purchased from GE Healthcare; all restriction endonucleases, Pfu DNA polymerase, T4 DNA ligase, and IPTG (isopropyl-β-D-thiogalactopyranoside) was from Fermentas. Oligonucleotides syntheses and sequencing services were provided by IDT DNA and 1^st^ BASE, respectively.

### Prediction of the interaction domains

The INT protein based on its amino acid sequence was modeled on I-TASSER server (http://zhanglab.ccmb.med.umich.edu/I-TASSER/). The interaction between MVP and INT was predicted by GRAMM-X protein docking web server (http://vakser.bioinformatics.ku.edu/resources/gramm/grammx). For the prediction, INT was selected as ligand for docking with CP-MVP as the receptor.

### Subcloning of the iMVP domains and INT in pET system

Histidine tag (6 × His-tag) was introduced to both INT and iMVP at their N-termini in vector pET-28a to facilitate purification. INT was subcloned between *Nde*I and *Xho*I restriction sites in vector pET-28a. For further study as reference, 15 amino acids at the C-terminus of His-INT were truncated. The designed oligonucleotides are listed on Supplementary Information Table S4. To isolate the MVP interaction domains, iMVP, DNA sequences containing domains 3, 4 and 5 (PDB ID 2QZV, residues 102–276) were PCR-amplified from the CP-MVP gene by Pfu polymerase and sub-cloned into pET-28a. The PCR products and the vectors were digested with restriction enzyme *Nde*I and *BamH*I then ligated. The mCherry genes were fused to the N terminal of INT (residues 1563–1724 of VPARP, GenBank accession No. AF158255) and cloned between *Nde*I and *BamH*I restriction sites on pET-11a.

### Gene expression and protein purification by chromatography

The cDNAs for iMVP domains, mCherry-INT and INT were over expressed in *E. coli* strain BL21(DE3). The production of mCherry-INT and His-INT was induced by 1 mM IPTG at 37 °C for 3 hours, and iMVP domains were induced by 0.1 mM IPTG at 20 °C for 16 hours. Cells were resuspended in binding buffer, Tris-HCl or phosphate buffered saline (PBS), and disrupted by sonication.

The purification of mCherry-INT was achieved by anion exchange chromatography (5 ml HiTrap Q FF column) on fast protein liquid chromatography (FPLC) ÄKTA-Explorer (GE Healthcare) system. The optimal binding buffer was determined to be 20 mM Tris-HCl at pH 7.4 and the mCherry-INT was eluted during linear gradient of NaCl (0.2–0.4 M).

His-tagged proteins were purified by affinity chromatography. His-iMVP domains and His-INT was purified on HisTrap HP column (1 ml) with binding buffer (50 mM Tris-HCl or 20 mM PBS, 150 mM NaCl and 25 mM imidazole, pH 7.4) and elution buffer (50 mM Tris-HCl or 20 mM PBS, 150 mM NaCl and 500 mM imidazole, pH 7.4). Typical peak fractions were collected for SDS-PAGE analysis. A full wavelength scan (from 200 nm to 800 nm) for mCherry-INT was performed with UV-VIS spectrophotometer (Shimadzu, UV-2450). The specific absorption wavelength of mCherry-INT was determined to be 585 nm which was used to monitor its presence during the chromatography to investigate the interaction between iMVP and INT.

All protein preparations for SPR experiments were subjected to size exclusion chromatography (SEC, GE Healthcare Superdex 75 10/300 GL column). Typical elution profile consisted of two peaks corresponding to dimer (D) and monomer (M) fractions. The M fractions were pooled and concentrated using ultrafiltration (Amicon, Milipore) and diluted as required.

All proteins were prepared fresh within 2 days and stored at 4 °C prior to experiments.

### Binding study using affinity chromatography

His-iMVP and mCherry-INT were mixed at equimolar molar ratio and incubated overnight at 4 °C before the affinity chromatography (1 ml, GE Healthcare HisTrap HP column). As control, mCherry-INT was loaded and found to flow through the column as unbound fraction.

### Binding study using size exclusion chromatography

His-iMVP and His-INT (assumed to be monomer during calculation) were mixed at equimolar molar ratio and incubated overnight at 4 °C before the SEC (GE Healthcare Superdex 75 10/300 GL column).

### Surface plasmon resonance (SPR) experiments

The Biacore 3000 system (GE Healthcare) was employed for SPR experiments. All experiments were performed at 25 °C. Replicated measurements were done for each sample injection (His-INTs at various concentrations; assumed to be monomer during calculation). His-iMVP was immobilized on the carboxymethyldextran-modified gold surface of sensor chip CM5 (GE Healthcare) by amine coupling at different densities in separate flow cells. N-hydroxysuccinimide (NHS) at 0.05 M/ethyl(dimethylaminopropyl) carbodiimide (EDC) at 0.2 M (v/v) was injected to activate the surface carboxylic groups to NHS esters. The NHS esters coupled with the primary amines on arginine and/or lysine residues in His-iMVP during the ligand injection. Unreacted NHS-esters were blocked by 1 M ethanolamine, pH 8.5. His-INT and variants were diluted as two-fold series in running buffer (50 mM PBS, 150 mM NaCl, pH 7.4), and injected at a flow rate of 30 μl/min across a blank surface and the immobilized His-iMVP surface for 120 s. Dissociation was monitored for 300 s in running buffer. Between each cycle, the chip surface was regenerated by 15 mM HCl for 30 s.

If the buffer pH of His-INT is lower than its isoelectric point (pI), His-INT will be generally positively charged, resulting in non-specific binding to the surface of sensor chip but not to His-iMVP. To remove these signals due to non-specific binding, one blank channel without ligand immobilized was used as a reference.

### Molecular dynamics simulations

All simulations and analyses were performed using the GROMACS5.0.4 simulation package^36–38^ with the CHARMM27 force field^39,40^. The structure and coordinates of proteins (INT, INT/DSA/3 H, and INTΔC15) were obtained from the Rosetta package (http://robetta.bakerlab.org). To produce the simulation condition of pH 6, the sidechain of His was modeled to be protonated, leading to a net charge of +1. A single protein was solvated with ~16,000 TIP3P-water molecules in a periodic box of size 8 nm/size, and then counterions (0~10 Cl^−^) and additional ions of 150 mM (46 Na^+^ and Cl^−^) were added. A pressure of 1 bar and a temperature of 298 K were maintained by applying a velocity-rescale thermostat^41^ and Parrinello-Rahman barostat^42^ in the NPT ensemble. A real space cutoff of 1.2 nm was applied for Lennard-Jones and electrostatic forces with the inclusion of particle mesh Ewald summation for long-range electrostatics^43^. The LINCS algorithm was used to constrain the bond lengths^44,45^. Simulations were carried out for 500 ns with a time step of 2 fs on computer facilities supported by the National Institute of Supercomputing and Networking/Korea Institute of Science and Technology Information with supercomputing resources including technical support (KSC-2017-C3-61). The last 200 ns was used for analyses.

## Electronic supplementary material

Supplementary Information
