# Supplementary material for: Modulation of the Vault Protein-Protein Interaction for Tuning of Molecular Release
Source: Sci Rep. 2017 Nov 1;7:14816. doi: 10.1038/s41598-017-12870-x (PMC5665922; doi:10.1038/s41598-017-12870-x)
Supplement: Supplementary file 1 — Supplementary Information [file 41598_2017_12870_MOESM1_ESM.pdf]

SUPPLEMENTARY INFORMATION

Modulation of the Vault Protein-Protein Interaction for Tuning of Molecular Release

*Kang Yu[1], Yin Hoe Yau[2], Ameya Sinha[1], Tabitha Tan[3], Valerie A. Kickhoefer[4], Leonard H. Rome[4,5], Hwankyu Lee[6], Susana Geifman Shochat[2], and Sierin Lim[1,7]**

[1] Bioengineering Division, School of Chemical and Biomedical Engineering; [2]Structural Biology and Biochemistry Division, School of Biological Sciences; [3]School of Materials Science and Engineering, Nanyang Technological University, 50 Nanyang Drive, Singapore 637457; [4]Department of Biological Chemistry, David Geffen School of Medicine at UCLA; [5]California NanoSystems Institute, University of California Los Angeles, Los Angeles, CA 90095, USA; [6]Department of Chemical Engineering, Dankook University, Jukjeon, Yongin, 448-701, South Korea; [7]NTU-Northwestern Institute for Nanomedicine, Nanyang Technological University, 50 Nanyang Drive, Singapore 637553

Corresponding Author

*Email: SLim@ntu.edu.sg

A 9-Å draft crystal structure of recombinant vaults was published by Anderson et al. (PDB ID 2QZV)[RS1]. The domain partitions from the Anderson's structure were used as a reference to identify the site of interaction between MVP and INT.

Table S1. MVP domain partitions[RS1].

Domain	Working Models (aa)	Origins of Starting Models
2	22-87 and 88-101	ROSETTA 1-87 and 88-112
3	113-166	NMR 1Y7X
4	167-221	NMR 1Y7X
5	222-276	Threaded onto Domain 4

Confirming interactions between isolated iMVP and INT

The interaction between purified His-iMVP and mCherry-INT was studied using affinity chromatography by monitoring the protein and mCherry absorbance at 280 and 585 nm, respectively. When individually loaded, only His-iMVP was bound to a HisTrap HP column while the mCherry-INT flowed through (Fig. S1A). Subsequently, His-iMVP and mCherry-INT at equimolar were mixed *in vitro* overnight at 4°C and loaded onto the affinity column. The co-elution of mCherry-INT and His-iMVP as indicated by the presence of peaks at both 280 nm and 585 nm during the elution step (Fig. S1B), suggested that the His-iMVP and mCherry-INT were interacting. The presence of both proteins in the co-elution fraction was confirmed by SDS-PAGE analysis (Fig. S1C). The observed protein peak in the flow-through fraction may be due to some impurities or unbound mCherry-INT. The results in this experiment confirm that INT binds to iMVP and that the N-terminal 6×His tag does not interfere with INT binding to iMVP.

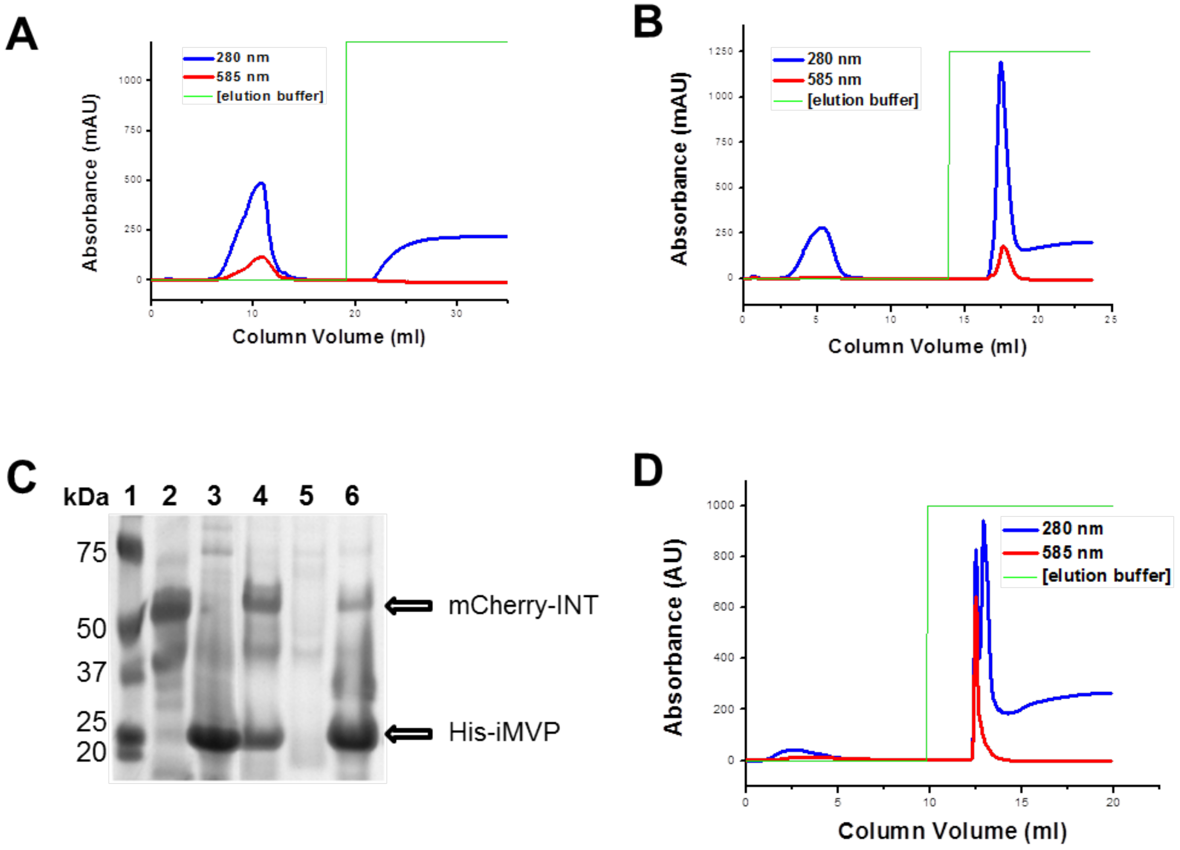

Figure S1. (A) Affinity chromatography elution profile of mCherry-INT as control showed mCherry-INT flowed through the HisTrap HP column without binding. (B) Affinity chromatography elution profile of His-iMVP and mCherry-INT mixture showed mCherry-INT was trapped in the HisTrap HP column due to interacting with His-iMVP. The elution buffer (green) came into the column together with 500 mM imidazole which was used to elute the trapped proteins. (C) SDS-PAGE of mixture (His-iMVP and mCherry-INT) peak from HisTrap HP column. 1: Protein molecular weight marker (Bio-Rad, Precision Plus Protein standards, all blue); 2: Semi-purified mCherry-INT; 3: His-iMVP; 4: Equimolar mixture of His-iMVP and mCherry-INT; 5: Flow through fraction; 6: Elute of bound fraction. (D) His-iMVP and mCherry-INT mixture result 2.

Fig. S1C (lane 4) shows three bands in the mixture of His-iMVP and semi-purified mCherry-INT prior to chromatography. Following coelution from affinity column, less mCherry-INT remained (lane 6) indicating that mCherry-INT interacts with His-iMVP. The higher density of His-iMVP present compared to mCherry-INT in the coeluted bound fraction maybe due to the

transient nature of the mCherry-INT and His-iMVP interactions. This is consistent with the variation observed in elution profile such as that shown in Fig. S1D. Unlike the single-peak coelution profile in Figure S1B, mCherry-INT and His-iMVP eluted in two distinct peaks with mCherry-INT eluted slightly earlier than His-iMVP at the elution stage. Despite the differential elution time, both proteins were interacting at the time of loading as shown by the binding to the column. Hence, the affinity between His-iMVP and mCherry-INT can be inferred confidently.

Monomer and dimer of His-INT

Figure S2 shows the full SDS-PAGE gel pictures for Figure 2 (B) and (C) for monomer and dimer of His-INT.

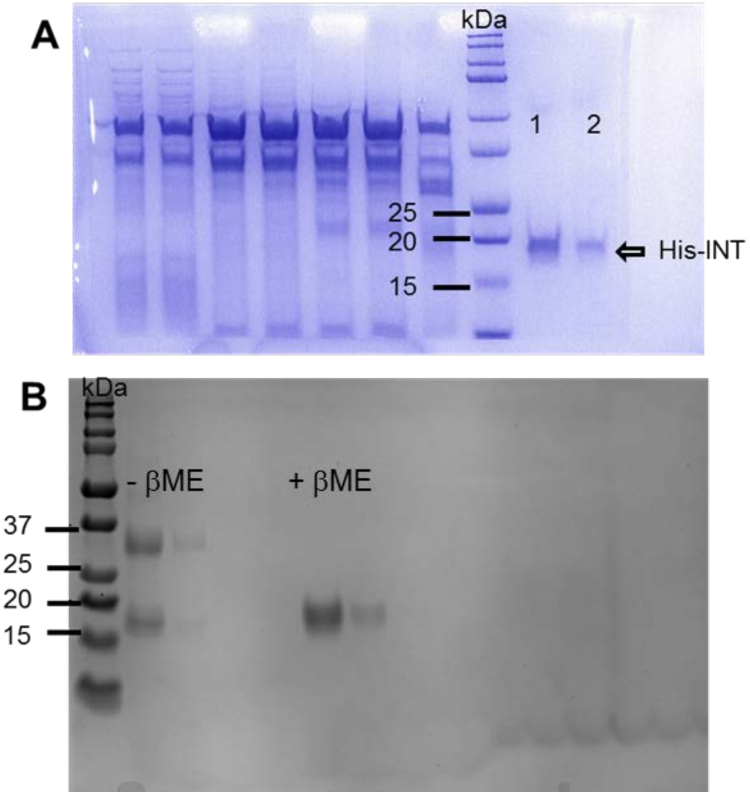

Figure S2. Full SDS-PAGE gel picture of Figures 2B (A) and 2C (B). Figure 2C image has been enhanced with +20% contrast.

Molecular mass determination of the size exclusion chromatography (SEC) fractions using MALDI-TOF/TOF mass spectrometry (MS), as well as SDS-PAGE of D fraction without and with β-mercaptoethanol (βME), suggest that His-INT also forms dimers (Figure S3). This observation may be due to the presence of three cysteine residues (GenBank accession No. AF158255; aa 1563, 1622 and 1687) on the His-INT. As shown, there was equilibrium between monomers and dimers.

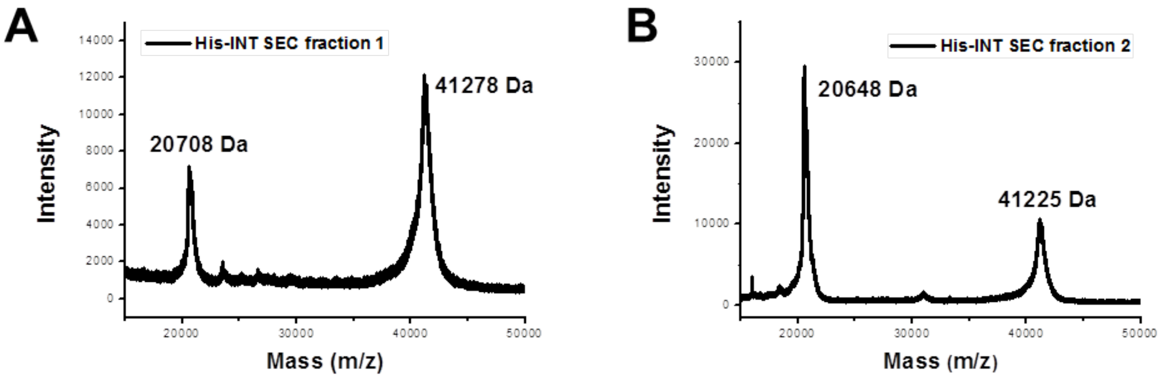

Figure S3. Profile of His-INT after SEC FPLC on MALDI-TOF/TOF Mass Spectrometry (MS). (A) fraction 1, (B) fraction 2.

Although monomers and dimers separated as two independent peaks in the SEC, the monomer peak fractions that were run a few hours after the SEC, showed again both species (Figure S4A, 10-15 ml). Complete separation of monomers from dimers is not achievable using the method. However, it is clear from Figure S4B that majority of the population in the "monomer" peak (fractions #14 and #15) is monomer. Figure S4C shows that the monomers and dimers are in thermodynamic equilibrium at the given concentration.

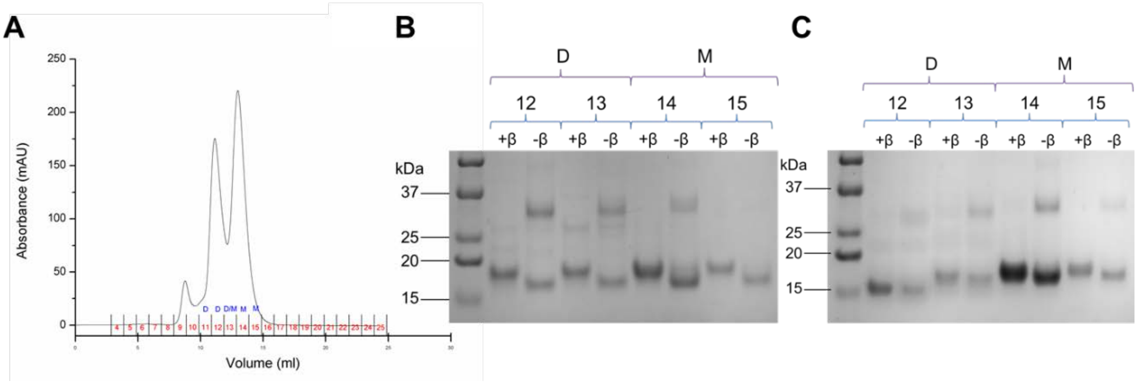

Figure S4. (A) SEC (Superdex 75) chromatogram of His-INT from His-affinity purified fractions, (B) SDS-PAGE of the SEC fractions in (A) with and without β-mercaptoethanol, (C) SDS-PAGE of the same fractions as in (B) after 19 days.

Supplementary analysis on SEC profile of equimolar mixture of His-iMVP and His-INT

Figure S5 and Table S2 show the analysis of peak fractions from SEC of mixture (Fig. 3A). The calculated molar ratio of fraction 5 (1:1.8) suggests that there is a covered peak of 1-to-2 interaction between His-iMVP and His-INT during elution volume. Fraction 6 shows basically equal amount of His-iMVP and His-INT, suggesting that the first obvious peak could be due to 1-to-1 complex. There are much more His-iMVP than His-INT in fraction 7, which means the second obvious peak should be single protein of His-iMVP. Single protein of His-INT and overlapping His-iMVP was shown in fraction 11.

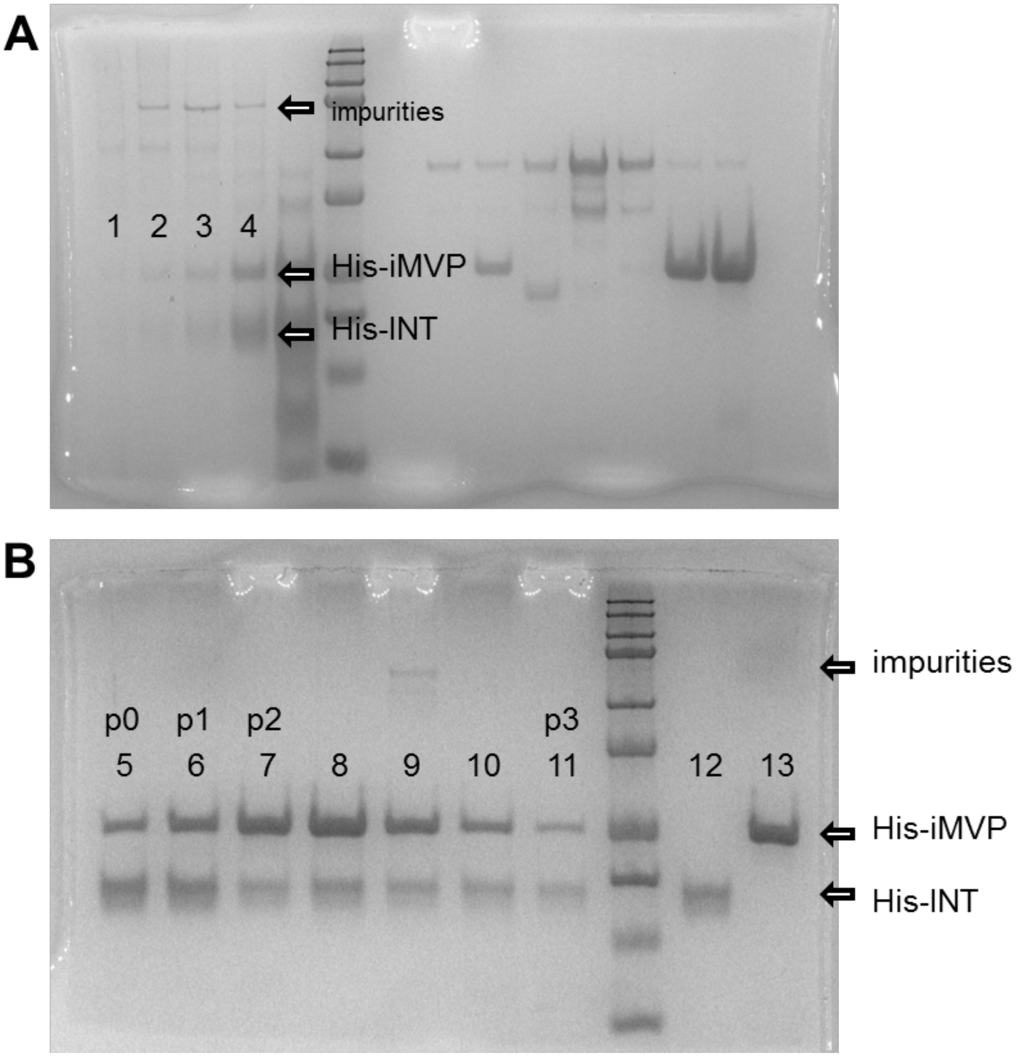

Figure S5. SDS-PAGE gel pictures of peak fractions from SEC in SEC profile of equimolar mixture of His-iMVP and His-INT in Figure 3A. (A) Fractions 1 to 4; (B) fractions 5 to 13. Figure 3B is redrawn using data extracted from (A) and (B).

Table S2. Fractions analysis of mixture SEC.

Elution Volume (ml)	Calculated MW (kDa)	Fraction in SDS-PAGE Gel	Molar Ratio Based on Band Density
(10.0, 10.5)	54.7	5	1:1.8
(10.5, 11.0)	45.2	6	1:1.0
(11.0, 11.5)	37.2	7	1:0.4
(13.0, 13.5)	17.2	11	1:1.1

Interaction between His-iMVP and His-INT at high immobilization density

The propensity toward 1:1 interaction in Fig. 4 predominates at low immobilization density (410 RU). In contrast, at high His-iMVP immobilization density (12,400 RU), the 1-to-1 model is no longer valid suggesting that more complicated interactions exist between the His-iMVP and the His-INT (Fig. S6). As shown in Fig. S6, as higher concentration of His-INT was injected, the binding would deviate farther from 1-to-1 and 1-to-2 fittings. Based on earlier observations of His-INT dimer and both 1:1 and 1:2 binding complex detected by SEC, the most probable interactions between His-iMVP and His-INT exist as both 1:1 and 1:2.

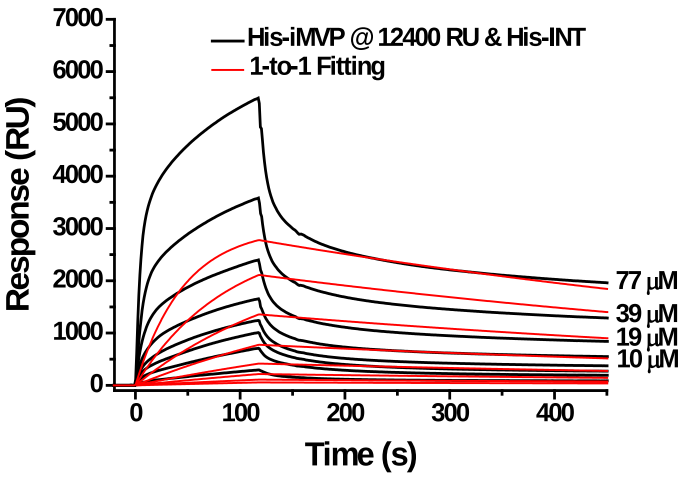

Figure S6. SPR sensorgram depicting the interaction between His-iMVP) (12400 RU) and His-INT. The lower four concentrations are 5, 2.5, 1.2, and 0.6 μM.

To understand the complex interactions at high immobilization density of His-iMVP, the interaction maps were analyzed by Ridgeview Diagnostics AB, shown as Fig. S7. At low immobilization density of His-iMVP, 1-to-1 interaction showed only one binding component. At high immobilization density of His-iMVP, two independent binding events were displayed. One

component occupied position near the heat map of 1-to-1 interaction. Considering the previous SEC and SDS analysis, the other component might correspond to 2-to-1 interaction.

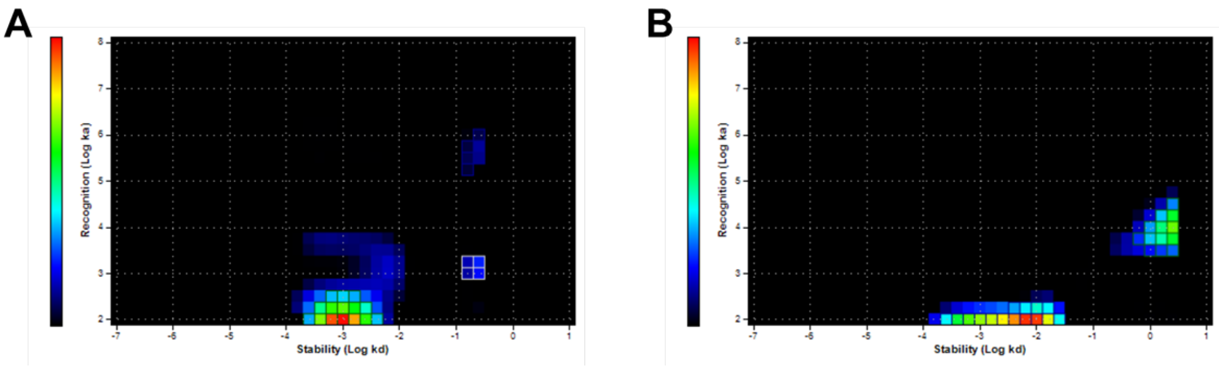

Figure S7. Interaction map analysis of two different densities of His-iMVP immobilization. His-INT: (A) 410 RU, (B) 12400 RU.

Mutant Design of INT

Table S3 summarizes that the key interaction sites on iMVP are negatively charged. Upon introduction of histidines on INT that are protonated and positively charged at acidic pH, the interaction is expected to be enhanced by electrostatic interaction.

Table S3. Side-chain charges of the key interaction sites on iMVP and INT.

iMVP	*INT*	*INT/DSA/3H*
209F	1694N	1694 N
210E(-)	1695 D(-)	1695 **H(+)**
211E(-)	1696 W	1696 W
212V	1697 D(-)	1697 D(-)
213L	1698 S	1698 **H(+)**
214D(-)	1699 A	1699 **H(+)**
215L	1700 T	1700 T
216V	1701 K(+)	1701 K(+)
217D(-)	1702 Q	1702 Q
218A	1703 L	1703 L

Interaction between His-iMVP and His-INT at pH 6.0

Figure S8 shows the SPR sensorgrams of His-iMVP and wild-type His-INT at pH 6.0. His-iMVP was immobilized at 12400 RU and His-INT was injected across the surface as a series of 0.44, 0.87, 1.75, 3.50, or 7.00 μM (assumed as monomer during calculation) in PBS buffer at pH 6.0, respectively. Although affinity constants could not be determined since the sensorgrams could not be fitted with a 1-to-1 Langmuir model, the observations at pH 6.0 seemed to fit bivalent fitting better suggesting 2 INT bind to 1 iMVP. Affinity constants of the bivalent binding was determined to be $k_{a1} = 1.03 \times 10^{4}$ $M^{-1}s^{-1}$, $k_{d1} = 0.279$ s^{-1}, $k_{a2} = 6.15 \times 10^{-6}$ RU^{-1} s^{-1}, and $k_{d2} = 8.26 \times 10^{-4}$ s^{-1}. It has to be noted that these numbers are mathematical representation which may not be compared with affinities of 1-to-1 binding.

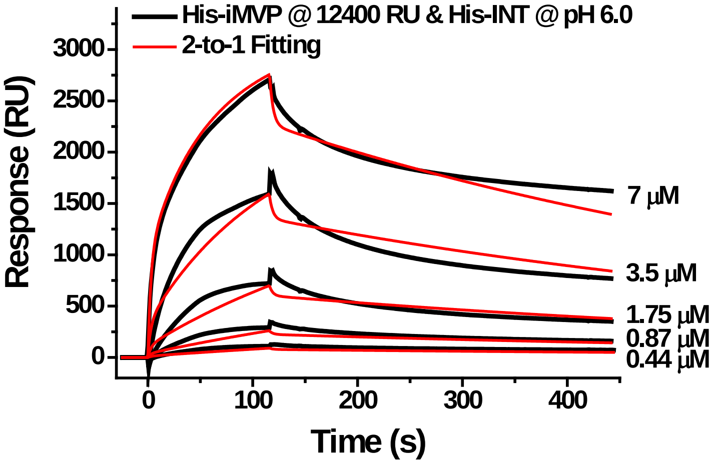

Figure S8. SPR sensograms of His-iMVP (immobilized phase) and mobile phase His-INT at pH 6.0.

Subcloning of the iMVP domains and INT in pET System

To isolate iMVP and construct INTs for investigations, the designed oligonucleotides are shown in Table S4.

Table S4. Oligonucleotides for isolated domains constructions.

Construct	Oligonucleotides*
His-iMVP	5’-ggaattccatatgGGGGAGGTGCTGGAAAAGGAC-3’ (forward)<br>5’-cgggatccttaCAGGGTGGTGATGGGTACTACCC-3’ (reverse)
mCherry-INT	5’-ggaattccatatgGTGAGCAAGGGCGAG-3’ (forward)<br>5’-cgggatccttaGCCTTGACTGTAATGGAGGACT-3’ (reverse)
His- INT	5’-gggaattccatatgTGCACACAACACTGGCA-3’ (forward)<br>5’-agctcgagttaGCCTTGACTGTAATGGA-3’ (reverse)
His-INTΔC15	5’-gggaattccatatgTGCACACAACACTGGCA-3’ (forward)<br>5’-atctcgagttaTATGGGCTGGAGTCCCAGCAA-3’ (reverse)

*Genes are in uppercase; restriction enzyme sites are underlined.

During the experiments, some other variants of MVP and INT were also investigated. The proteins produced are summarized as Table S5.

Table S5. Summary of truncated MVP and INT proteins production.

Protein variants	Description	Soluble for purification
His-MVP25	MVP domains 2, 3, 4, 5	No
His-MVP35	MVP domains 3, 4, 5	Yes
His-MVP34	MVP domains 3, 4	No
His-INTΔN15	15 amino acid deleted at the N terminus	No
His-INTΔC15	15 amino acid deleted at the C terminus	Yes
His-INTΔNC15	15 amino acid deleted at both of the N terminus and C terminus	No

REFERENCES

RS1 Anderson, D. H., Kickhoefer, V. A., Sievers, S. A., Rome, L. H. & Eisenberg, D. Draft Crystal Structure of the Vault Shell at 9-Angstrom Resolution. *PLoS. Biol.* **5**, 2661-2670 (2007).
